# Intermittent Cold Exposure Induces Distinct Proteomic Signatures in White Adipose Tissue of Mice

**DOI:** 10.3390/ijms26167898

**Published:** 2025-08-15

**Authors:** Elena Elsukova, Tatiana Zamay, Anna Kichkailo, Andrey Yakunenkov, Dmitry V. Veprintsev, Zoran Minic, Maxim V. Berezovski, Yury Glazyrin

**Affiliations:** 1 Department of Biology, Chemistry and Teaching Methods, Krasnoyarsk State Pedagogical University Named After V.P. Astafyev, Ady Lebedevoy 82, 660049 Krasnoyarsk, Russia; eielsukova@yandex.ru (E.E.); avy0905@yandex.ru (A.Y.); 2 Laboratory for Digital Controlled Drugs and Theranostics, Federal Research Center “Krasnoyarsk Science Center of the Siberian Branch of the Russian Academy of Sciences”, Akademgorodok 50, 660036 Krasnoyarsk, Russia; tzamay@yandex.ru (T.Z.); annazamay@yandex.ru (A.K.); d_veprintsev@mail.ru (D.V.V.); 3 Laboratory for Biomolecular and Medical Technologies, Prof. V.F. Voino-Yasenetsky Krasnoyarsk State Medical University, Partizana Zheleznyaka 1, 660022 Krasnoyarsk, Russia; 4 Department of Chemistry and Biomolecular Sciences, John L. Holmes Mass Spectrometry Core Facility, University of Ottawa, Ottawa, ON K1N 6N5, Canada; zminic@uottawa.ca (Z.M.); maxim.berezovski@uottawa.ca (M.V.B.)

**Keywords:** proteomics, adipose tissue, intermittent cold exposure, mitochondrial remodeling, thermogenesis, futile cycles

## Abstract

Adipose tissue exhibits dynamic metabolic and structural changes in response to environmental stimuli, including temperature fluctuations. While continuous cold exposure has been extensively studied, the molecular effects of prolonged intermittent cold exposure (ICE) remain poorly characterized. Here, we present a proteomic analysis of inguinal white adipose tissue (IWAT) from mice subjected to a 16-week regimen of short-term daily ICE (6 °C for 6 h, 5 days per week) without compensatory caloric intake. Mass spectrometry identified 1108 proteins, with 140 differentially expressed between experimental and control groups. ICE significantly upregulated mitochondrial proteins associated with lipid and carbohydrate catabolism, the tricarboxylic acid (TCA) cycle, oxidative phosphorylation, and lipogenesis, including LETM1, AIFM1, PHB, PHB2, ACOT2, NDUA9, and ATP5J. These changes reflect enhanced metabolic activity and mitochondrial remodeling. In contrast, proteins linked to oxidative stress, insulin resistance, inflammation, and extracellular matrix remodeling were downregulated, such as HMGB1, FETUA, SERPH1, RPN1, and AOC3. Notably, gamma-synuclein (SYUG), which inhibits lipolysis, was undetectable in ICE-treated samples. Our findings support the hypothesis that ICE promotes thermogenic reprogramming and metabolic rejuvenation in subcutaneous fat through activation of futile cycles and mitochondrial restructuring. This study offers molecular insights into adaptive thermogenesis and presents intermittent cold exposure as a potential strategy to mitigate adipose tissue aging.

## 1. Introduction

Adipose tissue, often called body fat, is a loose connective tissue mainly composed of fat cells (adipocytes). It has emerged as an organ that plays a key role in maintaining energy, metabolic, and temperature homeostasis, characterized by a complex cellular composition of various types of fat and non-fat cells that dynamically respond to external conditions [1]. The subcutaneous fat depot is noted for its remarkably complex cellular organization and high plasticity. Single-cell RNA sequencing has revealed seven subtypes of adipocytes in the inguinal subcutaneous depot [2] and only three subtypes of adipocytes in the epididymal visceral depot [3]. In recent years, special attention has been focused on beige-type adipocytes. These cells, like brown fat adipocytes, can undergo thermogenesis through the uncoupling protein UCP1, making them a potential therapeutic target in obesity treatment [4,5]. A significant decrease in UCP1 expression precedes age-related dysfunction, which occurs in the subcutaneous depot earlier than in other adipose tissues, leading to its gradual reduction [6]. Symptoms of aging are evident in the accumulation of hypertrophied metabolically inactive adipocytes, decreased insulin sensitivity, impaired adipogenesis, secretion of pro-inflammatory adipokines, and chronic low-grade inflammation [7,8]. These metabolic disorders are believed to contribute to the development of type II diabetes, hepatosteatosis, and cardiovascular diseases [7].

In experiments, maintaining animals at temperatures ranging from −4 to 4 °C, which stimulates beige adipogenesis and energy metabolism in subcutaneous fat, delays the symptoms of age-related dysfunction and systemic metabolic disorders [9]. Intermittent short-term exposures to cold, unlike constant adaptation to cold, are more physiological and carry fewer negative stress-related effects, making them potentially interesting for preventive medicine in the future. A positive impact of cold exposure courses initiated before puberty on adipogenesis in brown fat, the UCP1 expression in subcutaneous depots, as well as carbohydrate metabolism in mice, has been reported [10]. Theoretically, when adapting to periodic cold stimuli, temperature homeostasis may be better preserved through the swiftly responsive, low-inertia UCP1-dependent thermogenesis of beige adipocytes in the subcutaneous fat depot. In adult animals, particularly those with diet-induced obesity, the effectiveness of cold exposure might be completely or partially diminished due to increased food intake aimed at compensating for the energy costs of thermoregulation [11,12]. Consequently, combining cold exposure with moderate food restriction may be necessary to achieve a positive outcome [11].

To gain a deeper understanding of the mechanisms that slow down age-related dysfunction of adipose tissue, large-scale screening of biochemical processes using omics technologies is of interest. Researchers have studied the effect of continuously keeping animals at low temperatures (4–6 °C) for 3–18 days on the transcriptome of adipose tissues [13,14,15,16], as well as on individual adipocytes and non-fat cells [2]. Additionally, a proteomic analysis of subcutaneous adipose tissue was performed during a continuous 3-week period of cold adaptation in mice [17]. However, there is currently no information on the impact of prolonged intermittent cold adaptation on the transcriptome and proteome of adipose tissues. The purpose of our study was to analyze changes in the proteome of the subcutaneous fat depot of laboratory mice subjected to prolonged intermittent short-term cold exposures without energy compensation for their costs. The discovery of the molecular mechanisms of slowing down age-related dysfunction of adipose tissue using low-temperature exposure is important for a scientifically based approach to the prevention and treatment of age-related diseases.

## 2. Results

**Figure 1:**
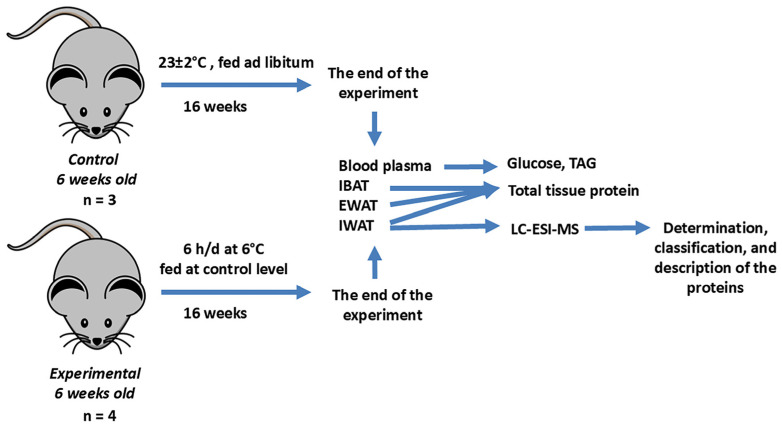
The animal study design. IBAT—interscapular brown adipose tissue, EWAT—epididymal white adipose tissue, IWAT—inguinal white adipose tissue, TAG—triacylglycerol, LC-ESI-MS—liquid chromatography–electrospray ionization–mass spectrometry.

The animal study design is shown in Figure 1. After a 1.5-week adaptation period at a temperature of 23 ± 2 °C and a balanced pelleted diet, seven mice were divided into two groups. The control group continued under normal dietary and temperature conditions, and the experimental group was exposed to 6 °C for 6 h each day, 5 days a week, over a span of 16 weeks, with a food amount equivalent to the control group. At the end of the experiment, animals were sacrificed, the concentrations of glucose and triglycerides in blood plasma were determined, and total tissue proteins of the interscapular brown adipose tissue (IBAT), the epididymal white adipose tissue (EWAT), and the inguinal white adipose tissue (IWAT) were measured. IWAT samples from three control and four experimental mice were selected for proteomic analysis.

### 2.1. Effect of Intermittent Cold Exposures on Body Weight and Adipose Tissues

**Figure 2:**
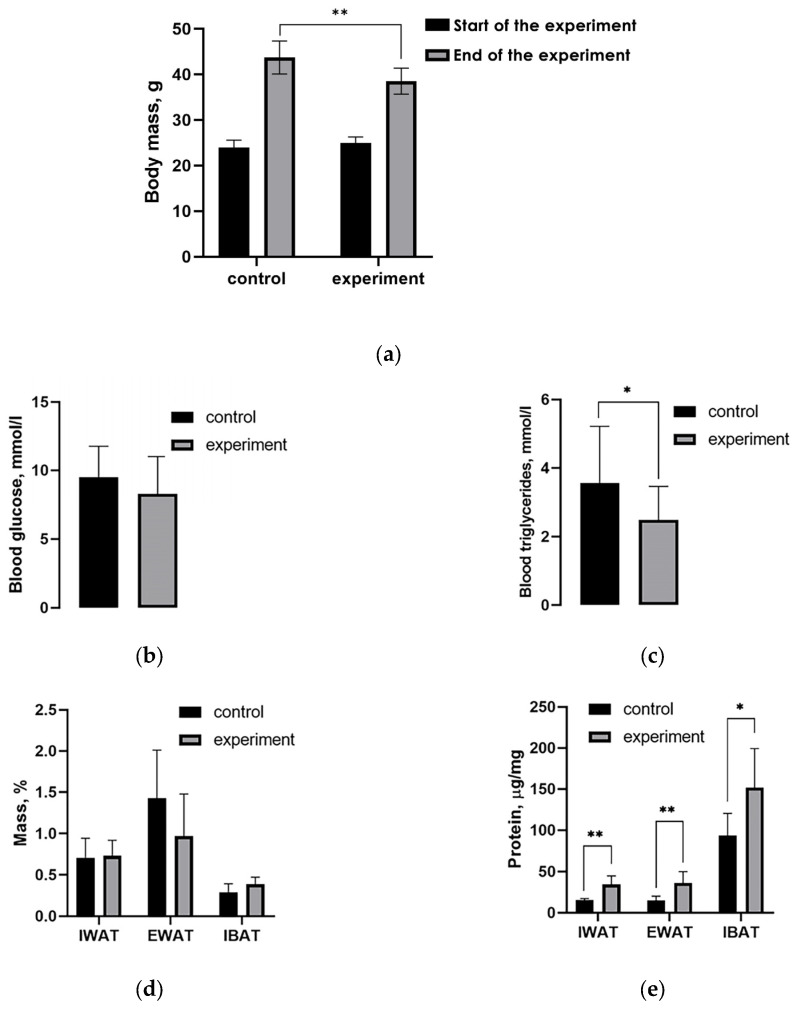
The effect of intermittent cold exposures on body weight; blood glucose and triglyceride levels; adipose tissues mass; and protein content. (**a**) Body weight; (**b**) blood glucose; (**c**) blood triglycerides; (**d**) relative adipose tissue mass; (**e**) total protein content in adipose tissue. * *p* < 0.05, ** *p* < 0.01, Mann–Whitney U test.

During the intermittent cold exposure (ICE) experiment, the average body weight of mice increased by 1.9 and 1.5 times in the control and experimental groups, respectively. Consequently, in the experimental group, this measure was 15.5% lower than in the control group at the end of the experiment (Figure 2a). At the end of the experiment, the average values of blood glucose and triglyceride were 13% and 25% lower in the experimental group of mice compared with the control group (Figure 2b,c). The experimental group of mice also exhibited a 1.5-fold decrease in the relative weight of EWAT compared with the control group (Figure 2d). The relative masses of IWAT and IBAT were nearly identical. Differences in blood glucose between animal groups were not statistically significant. The relative content of total protein (µg/mg) in all three tissues of the experimental mice was higher than that in the control mice (Figure 2e). The increase in the relative protein content depends on several factors. Firstly, the deposition of lipids in adipocytes decreases due to a change in the balance of lipogenesis and lipolysis processes in favor of the latter, which is accompanied by a decrease in the size of lipid droplets. Noradrenergic activation of lipolysis provides cold-induced thermogenesis in IBAT with fatty acids; adipocytes of fat depots export fatty acids in IBAT and other tissues involved in cold adaptation [1] into inducible beige adipocytes in the depots themselves. Secondly, the activation of lipolysis, beta-oxidation, and thermogenesis in adipose tissues should inevitably be accompanied by an increase in the synthesis of enzymes and other proteins related to mitochondriogenesis; these processes are collectively called “metabolic remodeling”. In IBAT, the processes of adaptive remodeling of adipocytes develop especially actively in mice during adaptation from the thermoneutral range to 20 °C; when temperatures decrease below 20 °C, an additional contribution to the increase in thermogenic reserve is made by increased cellular proliferation and suppression of apoptosis, which is accompanied by an increase in tissue mass [18]. In white adipose tissue, the processes of metabolic remodeling, such as beige adipogenesis, appear at temperatures below 20 °C; no significant changes in the content or ratio of adipocytes, their precursors, and non-adipocyte cells were observed [2].

### 2.2. Proteomic Analysis of IWAT

**Figure 3:**
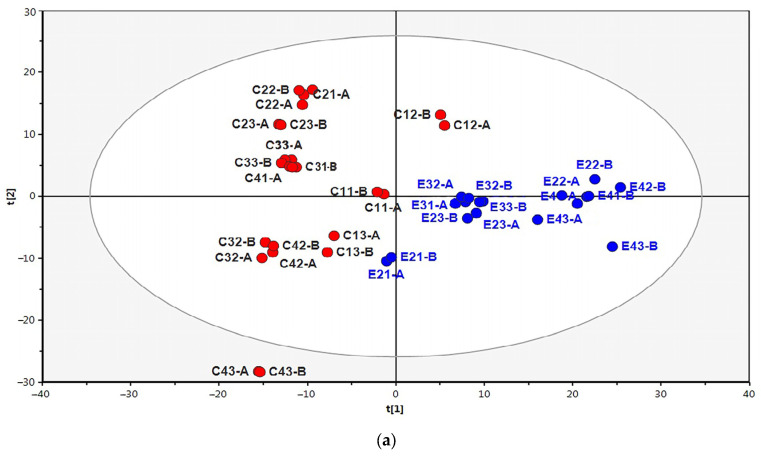

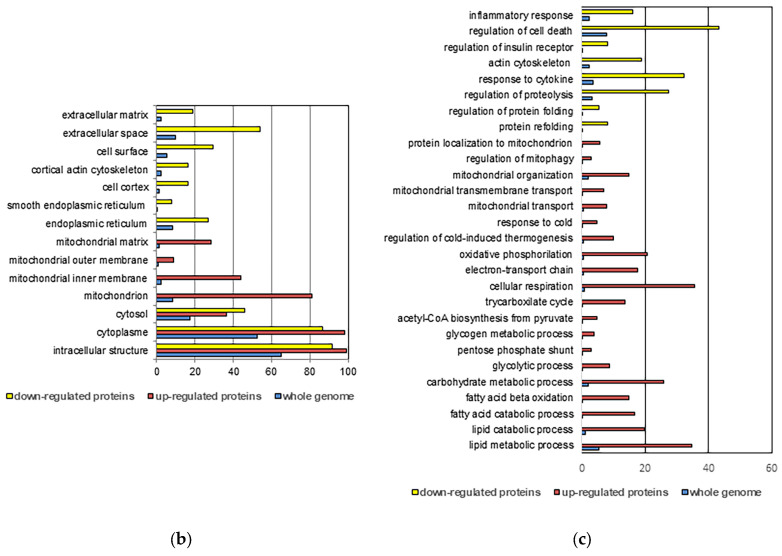
The effect of intermittent cold exposures on proteome of inguinal adipose tissue. (**a**) Principal component analysis diagram, where red and blue circles represent control and experimental samples, respectively. Overrepresentation test for differentially expressed proteins and total gene products in the Cellular Components (**b**) and Biological Processes (**c**) Gen Ontology (GO) categories.

Mass spectrometry identified 1108 proteins in the IWAT samples. Principal component analysis was used to visualize the variance in protein expression between control and experimental samples. In the diagram, control and experimental samples were clearly separated and clustered into two distinct groups (Figure 3a), indicating that the intermittent cold exposure regimen significantly impacted protein expression in IWAT. The main marker of beige adipocytes, the uncoupling protein UCP1, was present in 25% of the samples from the experimental group of mice and was not detected in any of the samples from the control group. Differential expression analysis revealed 140 proteins. Among these, 96 proteins had LFQ values that were 1.4 to 15 times higher in experimental samples, along with 6 proteins found only in these samples, which were classified as up-regulated proteins (Table 1). Additionally, 36 proteins showed LFQ values that were 1.4 to 17 times lower in experimental samples—along with gamma-synuclein, which was only detected in control samples—and were categorized as down-regulated proteins (Table 2).

#### 2.2.1. Functional Analysis of Up-Regulated Proteins

Analysis using Gen Ontology (GO) for cellular components [19] showed that 81.37% of up-regulated proteins were attributed to mitochondria, including 51% of mitochondrial membrane proteins and 28.43% of mitochondrial matrix proteins (Figure 3b). According to the overrepresentation test, the proportion of gene products in the Mitochondrion category was 9.48 times higher in the up-regulated proteins list than in the entire mouse genome. The classification of proteins using the GO for biological processes demonstrated the predominance of metabolic enzymes among up-regulated proteins (Figure 3c). The proportion of gene products in the Cellular Respiration, Lipid Metabolism, and Carbohydrate Metabolism categories was 44.55, 6.23, and 12.87 times higher than in the entire mouse genome. The Cellular Respiration category included subunits of the respiratory complexes of the electron transport chain (ETC) and ATP synthase, as well as enzymes of the pyruvate dehydrogenase complex and the tricarboxylic acid cycle (TCA). Proteins of the ETC accounted for 17.82% compared to 0.45% in the entire genome, and together with ATP synthase subunits (oxidative phosphorylation group), they made up 20.79% versus 0.48% in the whole genome. TCA cycle enzymes accounted for 13.86%, while in the entire genome their share was only 0.15%.

In the overlapping categories of Lipid Metabolism, Lipid Metabolism Processes, and Fatty Acid Metabolism Processes, enzymes from catabolic pathways predominated. These pathways include lipolysis, beta-oxidation of fatty acids, oxidation of unsaturated and polyunsaturated fatty acids, and the oxidation of fatty acids with an odd number of carbon atoms. Anabolic lipid metabolism pathways were represented by enzymes involved in fatty acid and triglyceride synthesis; enzymes that supply acetyl residues (ACLY, ACSA, and CACP); the enzyme ECHD1, which corrects accidental carboxylation of propionyl and butyryl residues of ACACA [20]; and the enzyme G6PD1 from the pentose phosphate pathway, which supplies NADPH for lipid biosynthesis. Enzymes of the citrate–malate shuttle, which also generate NADPH, were found in the NADP Metabolic Process category. Other proteins in the Lipid Metabolism category included enzymes that replenished the pools of oxaloacetate, acetyl, and succinyl residues (PIC and CAH5B; ODBB) in the TCA cycle; fatty acid transporters in the cytoplasm and nucleus (FABP5); the transcription factor (THRSP); and the lipid droplet membrane protein (PLIN1). In the category of Carbohydrate Metabolic Process, there were enzymes involved in glycogen metabolism, glycolysis, the pyruvate dehydrogenase complex, and the pentose phosphate pathway.

Several overlapping categories are associated with transport across the mitochondrial membrane and with processes that maintain the structural integrity of mitochondria. In the Mitochondrial Transport and Mitochondrial Transmembrane Transport categories, there are tricarboxylate transporter (TXTP); carnitine fatty acid transporter (MCAT); ADP/ATP translocases (ADT1 and ADT2); H^+^-dependent transporter of K^+^ and Ca^2+^ (LETM1) [21]; apoptosis-inducing factor 1 (AIFM1) and 60 kDa heat shock protein (CH60), which both control the import of proteins through the inner membrane [22]; and a potential-dependent anion channel (VDAC1) in the outer membrane. Hexokinase 2 is classified as a membrane protein, apparently due to its localization on the outer mitochondrial membrane, where it interacts with VDAC1 [23]. The Mitochondrial Organization category includes many proteins from the two previous categories (AIFM1, CH60, LETM1, ADT1, ADT2, HXK2); the scaffolding proteins PHB and PHB2; some accessory subunits of respiratory complexes (NDUS1, NDUS3, NDUAA, NDUA9) involved in the correct assembly and functioning of the ETC; antioxidant enzyme thioredoxin reductase (PRDX3); and outer membrane insertase (MTCH2) [24]. MTCH2, together with the lipogenesis intermediate lysophosphatidic acid, also initiates mitochondrial fusion processes, accelerating and increasing the efficiency of energy transport in the cell [25].

Analysis of LFQ intensities revealed that a significant increase in expression was most frequently observed in mitochondrial proteins. The PHB, PHB2, AIFM1, LETM1, accessory subunit of respiratory complex I (NDUA9), Rieske subunit of respiratory complex III (UCRI), and enzymes ACOT2 and CAH5B exhibited more than a 10-fold increase in LFQ. Among non-mitochondrial proteins, a fourteen-fold increase in expression was noted for GLGB, one of the glycogen synthesis enzymes. Six proteins were detected only in the experimental group, including the first rate-limiting lipolysis enzyme, adipose triglyceride lipase (PLPL2, also known as ATGL), and five proteins of the inner mitochondrial membrane (NDUAA, MCAT, ADT1, MTCH2, and ODBB).

#### 2.2.2. Functional Analysis of Down-Regulated Proteins

Analysis using GO for cellular components showed that proteins with reduced expression were distributed in the cytosol (45.95%), in the endoplasmic reticulum (EPR) (27%), in the cortical actin layer (16.22%), on the cell surface (29.73%), in extracellular space (54.05%), and in the extracellular matrix (18,92%) (Figure 3b). The representation of proteins in these categories was 2.6, 3.2, 28, 5.6, and 5.3 times higher than in the whole genome. Some proteins, such as HMGB1, FETUA, and PPIA, were present in several cell compartments and in the extracellular matrix (ECM). In the GO analysis of biological processes, several overlapping broad categories were identified, including Biological Regulation; Response to Stimulus; Cellular Component Organization or Biogenesis; and Macromolecule Metabolic Process, which were divided into subcategories (Figure 3c). Cytoplasmic proteins were primarily represented in subcategories such as Actin Cytoskeleton, Protein Folding, and Organelle Organization. Extracellular proteins were predominant in the Transport, Iron Ion Transport, Vesicle-Mediated Transport, and Immune System Process subcategories.

Gamma-synuclein attracts significant attention among cytosolic proteins because it was presented in all control samples and was not detected in any of the experimental samples. The function of this protein in adipocytes is not well understood. It was found [26] that gamma-synuclein is nutritionally regulated and increased in obesity. HFD-fed gamma-synuclein–null mutant mice are protected from obesity due to increased lipolysis and lipid oxidation. Gamma-synuclein knockdown in adipocytes causes redistribution of the key lipolytic enzyme ATGL to lipid droplets and reduces the content of SNARE-complexes involved in their fusion. Therefore, this protein was suggested to promote the fusion of lipid droplets and inhibit lipolysis, preventing the accumulation of ATGL on lipid droplets [26]. EPR proteins included chaperones involved in the folding of extracellular matrix glycoproteins (RPN), collagens (SERPH1), integrins, and Toll receptors (ENPL) [27,28]. The greatest ten-fold decrease in the LFQ was observed for riboforin (RPN), which, as part of the oligosaccharide transferase complex, interacts with non-folded or incorrectly folded glycoproteins, delaying their transport and contributing to their folding [28]. A decrease of more than three-fold was noted for serpin H1 (SERPH), which prevents local unfolding and aggregation of collagen fibrils [27]. Serine protease inhibitors (A1AT2 and A1AT3), known to suppress stress-induced synthesis of proinflammatory cytokines [29], showed a 2.96- and 2.66-fold reduction in LFQ, respectively. Nuclear proteins that showed down-regulation included nucleolin (NUCL), which is involved in transcription and maturation of ribosomal RNA; high mobility protein box 1 (HMGB1), involved in chromatin remodeling processes [30]; and heterogeneous nuclear ribonucleoprotein A2/B1 (ROA2), a coactivator of transcription factors in response to oxidative stress [31]. HMGB1 demonstrated the greatest decrease in LFQ by 3.6 times in the nuclear protein group. It should be noted that the localization of HMGB1 in the adipocyte depends on the cellular status. Nuclear HMGB1 can translocate into the cytosol and can be passively or actively released by adipocytes from obese mice and humans [32]. Among the plasma membrane proteins, attention is drawn to the guanine nucleotide-binding protein G(i) alpha-2 (GNAI2) and semicarbazide-sensitive amino oxidase (AOC3). GNAI2 functions in anti-lipolytic signaling, and its deficiency is accompanied by resistance to diet-induced obesity [33]. AOC3, on the outer surface of the adipocyte membrane, oxidizes endogenous and exogenous amines to form aldehydes, ammonium ions, and H_2_O_2_. H_2_O_2_ stimulates phosphorylation of insulin receptor substrate proteins and mimics insulin effects, such as GLUT4 translocation and lipolysis inhibition [34].

The expression levels of albumin (ALB), the proteoglycan lumican (LUM), and the secreted factors (F13A and FETUA) decreased in the extracellular matrix (ECM). All these proteins are recognized as markers of ECM remodeling linked to excessive intake of palmitate and glucose by adipocytes, contributing to the development of obesity along with insulin resistance [35]. Factor XIII-A transglutaminase (F13A) catalyzes the formation of cross-glutamyl-lysine covalent bonds in fibronectin, facilitating its accumulation from plasma, leading to collagen deposition, inhibition of preadipocyte differentiation, and attraction of pro-inflammatory macrophages [36]. Albumin protects against irreversible changes in fibronectin conformation caused by mechanical pressure on the matrix from hypertrophied adipocytes [37]. The proteoglycan lumican enhances the collagen hydrogel’s resistance to compressive forces [38]. Fetuin A (FETUA) interacts with the beta subunit of the insulin receptor, inhibiting insulin-dependent glucose transport. Through various receptors and signaling pathways, it suppresses mitochondriogenesis and fatty acid synthesis by reducing the expression of PPARγ and adiponectin, while promoting the incorporation of fatty acids and lipogenesis [39]. FETUA and HMGB1 trigger inflammatory processes by directly recruiting M1-type inflammatory macrophages and by transforming M2-type macrophages into inflammatory M1-type macrophages [32,39]. PPIA autocrinally stimulates the expression of key adipogenic transcription factors [40].

The Extracellular Space category included mainly proteins that provide transport of fatty acids (albumin, APOA4), vitamin D (VDBP), iron (HEMO, CERU, TRFE), and the heavy chain of immunoglobulin M. Ceruloplasmin, oxidizing Fe^2+^ to Fe^3+^, and IgM heavy chain showed the especially significant LFQ reduction by 10 and 16 times, respectively.

## 3. Discussion

In our work, the proteome of subcutaneous white adipose tissue was studied for the first time in mice after a long course of regular cold exposures without cold-induced hyperphagia. Our study was a trial and therefore may have a number of limitations. A significant one is the small sample size, and validation on larger cohorts will be required to improve the reliability and reproducibility of the results obtained. In addition, the hypothetical model of metabolic remodeling of subcutaneous adipose tissue adipocytes during cold exposures, constructed on the basis of the results of proteomic analysis, has not yet been confirmed by functional studies, such as basal mitochondrial respiration on different substrates to assess the contribution of carbohydrate and lipid oxidation to energy metabolism and heat production; oligomycin-sensitive respiration of mitochondria and adipocytes to detect mitochondrial uncoupling; and determination of the intensity of lipolysis and lipogenesis, adipocyte size, lipid droplet area, etc. In addition, due to the low content of beige adipocytes in white adipose tissue even with chronic cold adaptation, the results of mass spectrometric determination of UCP1 should be supplemented with sensitive immunochemical methods.

It is assumed that the studied intermittent cold adaptation regimen can inhibit age-related metabolic disorders manifested in obesity, insulin resistance, type II diabetes mellitus, non-alcoholic liver disease, etc. However, this assumption is also not yet sufficiently supported by all the necessary analyses and functional tests. In particular, determination of basal blood glucose is not an informative enough analysis for assessing glucose homeostasis; it is necessary to include functional analyses such as insulin tolerance and glucose tolerance tests. It is desirable to determine the level of triglycerides not only in the blood, but also in the liver.

When cold exposure is combined with limited food supply, as in our experiment, it is of interest to monitor the intensity of energy metabolism and body temperature to identify hypometabolic states in experimental animals.

The control group of animals in our experiment was kept at a temperature of 23 °C, which is below the thermoneutral zone of mice (>28 °C). It could lead to an underestimation of the changes induced by regular cold exposures in the experimental animals. Although it was shown in the study of Kalinovich et al. [18] that at 21 °C there are no thermogenic beige adipocytes in the subcutaneous depot, the expression of the UCP1 protein is detected only with a further decrease in temperature to 5–10 °C. The maximum content of UCP1 in mitochondria is achieved in IBAT cells at 21 °C compared to 30 °C, and thermogenesis is increased; therefore, we cannot exclude that white adipose tissue, including the subcutaneous depot, can, for example, enhance lipolysis for the export of fatty acids for the thermogenic needs of IBAT.

In this study, we were interested in whether long-term intermittent cold exposure without compensation of energy expenditure could lead to changes in the subcutaneous adipose tissue proteome that counteract age-related metabolic disorders. We found that a 3.5-month regimen of 6 h exposure at 6 °C (5 days/week) suppressed the age-related downregulation of UCP1 expression, as well as increased the expression of enzymes and other proteins involved in lipid and carbohydrate catabolism, the TCA cycle, the ETC, and de novo fatty acid synthesis and lipogenesis. In contrast, proteins that mark oxidative stress, insulin resistance, obesity, and inflammation showed reduced expression. The detected proteomic changes generally correlate positively with the previously studied dynamics of the transcriptome and proteome under various regimes of continuous cold adaptation [13,14,15,16,17].

**Figure 4:**
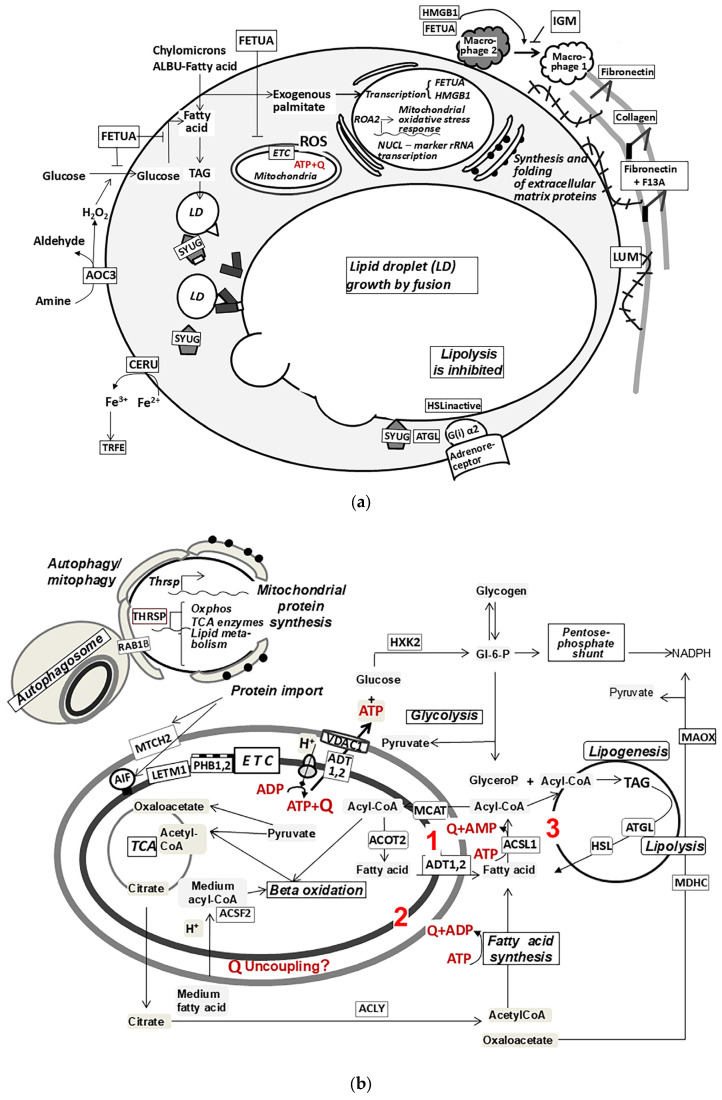
Schematic representation of an adipocyte of mouse subcutaneous adipose tissue under standard and experimental conditions. (**a**) Under standard conditions, the use of exogenous fatty acids for lipogenesis is increased, while insulin-dependent glucose transport, de novo fatty acid synthesis, and mitochondriogenesis are suppressed. There is a fusion of lipid droplets into one drop and the growth of it and the whole cell. Compensatory enlargement in the size and stiffness of the extracellular matrix and increased expression of proteins involved in danger signaling favor the accumulation of inflammatory macrophages; in contrast, IgM synthesis suppresses inflammation. Oxidative stress slows down due to the removal of Fe^2+^ from tissues by a system of ceruloplasmin and transferrin. (**b**) Under experimental conditions, the adipocyte synthesizes fatty acids and triglycerides de novo. For the uninterrupted supply of glucose and ATP to these processes, glycogen deposition and expression of HXK2, which intercepts ATP on the outer membrane of mitochondria, are enhanced. The fatty acids released during lipolysis are directed to the beta oxidation pathway. A part of the acyl-CoA is hydrolyzed by the mitochondrial enzyme ACOT2 to free fatty acids (1). This and the other futile cycles—fatty acid synthesis-beta oxidation (2), lipogenesis–lipolysis (3), as well as, probably, the mitochondrial uncoupling due to carnitine-independent transport of medium-chain fatty acids—intensify energy metabolism. To maintain the high biological quality of mitochondria, mitochondriogenesis and autophagy–mitophagy processes are enhanced.

The analysis of the obtained data provides a hypothetical sequence of events regarding the metabolic and structural remodeling of inguinal adipose tissue under temperature adaptations. At standard temperature conditions, once differentiation is completed, adipocytes enter the stage of lipid expansion (Figure 4a). This is a normal phase for subcutaneous adipose tissue, which serves as a strategic depot of TAG. According to our data, accelerated lipogenesis at this stage is facilitated by the increasing use of fatty acids transported for TAG synthesis by chylomicrons from outside the cells. The actively expressed gamma-synuclein reorganizes the TAG depot, ensuring that many small lipid droplets with a large total surface area for enzymes fuse into one growing lipid droplet [26]. Furthermore, the mobilization of lipolysis is hindered by synuclein-promoted disruption of the interaction of triglyceride lipase with a lipid droplet and a decrease in cAMP due to increased expression of the alpha-2 subunit of the Gi protein. Exogenous palmitate has previously been shown to stimulate the expression and secretion of alpha-fetuin [41]. In particular, alpha-fetuin inhibits insulin-dependent glucose transport, carbohydrate-based lipogenesis, and mitochondriogenesis in adipocytes [39]. Inhibiting de novo fatty acid synthesis is advisable when there is sufficient intake of fatty acids from the blood, since this process requires high energy costs. Consequently, this energy can be redistributed to cellular growth processes. Additionally, reducing ATP demand, and thus the need for mitochondria and mitochondriogenesis, allows for more cellular space to be available for the growing lipid droplet. However, substrate overload of the mitochondrial ETC increases the production of reactive oxygen species [42]. Other sources of reactive oxygen species include enzymatic reactions that produce hydrogen peroxide, which reacts with divalent iron to form a hydroxyl radical. These reactions are catalyzed by protein disulfide isomerases and amine oxidase AOC3. In contrast, the high levels of iron transport proteins (hemopexin, transferrin) and particularly the ferrooxidaseceruloplasmin, which oxidizes Fe^2+^ to Fe^3+^, can be regarded as a protective mechanism against free radical oxidation induced by free heme and Fe^2+^ [43].

In addition to oxidative stress and insulin resistance, adipocytes also experience mechanical stress. The growing lipid droplet exerts mechanical pressure on the cytoplasm and plasma membrane. Mechanical forces from hypertrophied adipocytes contribute to the pathological remodeling of the ECM by coagulation factor XIII and lumican. The enhanced volume and stiffness of the ECM interfere with adipocyte growth and the maturation of preadipocytes. The state of cellular stress in the inguinal adipose tissue of control mice is indicated by increased expression of HMGB1. It is considered a universal stress modulator and extracellularly acts as a damage-related molecular pattern that activates cytokine secretion by macrophages, contributing to chronic inflammation in adipose tissue [32,44]. Enhanced ECM density and stiffness are also believed to implicate the development of insulin resistance, the attraction of leukocytes, especially macrophages, and their polarization into the inflammatory M1 type [45]. Leukocytes with anti-inflammatory activity have also been identified in adipose tissues. These include regulatory T-lymphocytes and B-1 lymphocytes that produce natural immunoglobulin M. A significant increase in the expression of the IgM heavy chain indicates the presence of B1 lymphocytes in the samples from control mice, which can restrain the inflammatory process in subcutaneous adipose tissue [46].

Thus, the transition of a mature adipocyte to a strategy that reduces fatty acid synthesis, increases fatty acid transport into the cell from circulation, and favors deposition over lipolysis triggers a series of ongoing metabolic and regulatory manifestations of cellular aging. Interestingly, a similar course of events was discovered through single-cell RNA sequencing during the induction of obesity by a high-fat diet. In the epididymal adipose tissue of mice, a subpopulation of adipocytes responsible for synthesizing fatty acids disappeared, while the relative quantity of adipocytes that scavenge fatty acids from the environment and those expressing stress proteins increased [3]. This supports the perspective on the similarity between the cellular mechanisms of aging and obesity, considering obesity as a form of accelerated aging [7].

Before the study, it was assumed that cold exposure would slow the age-related loss of beige adipocytes in the inguinal fat depot. The fast-acting, low-inertia UCP1-dependent thermogenesis of these cells serves as a more favorable mechanism for maintaining temperature homeostasis in the lower limbs during intermittent short-term cold exposures. Thermogenesis in beige adipocytes and brown fat reduces the influx of energy substrates into white adipocytes for the synthesis and deposition of TAG. The visually noticeable darkening of inguinal fat, coupled with an increase in the expression of lipolysis enzymes and mitochondrial proteins related to the beta-oxidation of fatty acids, TCA cycle, and ETC, appears to support this hypothesis. The most significant increase in expression was observed for subunits of respiratory complexes, transport proteins of the inner mitochondrial membrane, and scaffold proteins of prohibitins I and II, which ensure proper assembly and stabilize respiratory complexes. The primary marker of beige adipocytes, the uncoupling protein UCP1, was detected in only 25% of the experimental samples. The expression levels of ATP synthase subunits and ADP/ATP translocase 1 and 2 were elevated in all samples. Consequently, the reorganization of mitochondria was largely attributed to an increase in ATP-dependent processes (Figure 4b).

The main ATP-dependent process in subcutaneous adipocytes is the synthesis and deposition of TAG, which maintains a strategic reserve of energy resources and provides thermal insulation for the body. Under regular cold exposure, competition between white adipocytes and brown fat for circulating fatty acids increases [47]. As a result, white adipocytes must synthesize fatty acids from carbohydrates and amino acids. Indeed, in the experimental samples, the expression of enzymes involved in the fatty acid synthesis pathway, glycolysis, citrate–malate shunt, branched amino acid oxidation, the oxidative branch of the pentose phosphate pathway, and cytoplasmic glycerophosphate dehydrogenase increased. The predominant distribution of ATP in the carbohydrate metabolism pathways associated with fatty acid synthesis is achieved by raising the expression of hexokinase 2. This isoenzyme, located on the outer membrane of mitochondria, quickly intercepts ATP that is transported to the cytoplasm through the outer membrane by the VDAC-1 channel [23]. The accumulation of glycogen in adipocytes ensures a continuous supply of glucose for lipid synthesis. This is supported by our data showing a significant increase in the expression of glycogenesis enzymes and, to a lesser extent, glycogen phosphorylase expression. Furthermore, early observations indicate that glycogen deposition precedes lipogenesis when transitioning from calorie-restricted diets to ad libitum nutrition [48]. Additionally, increased expression of the glycogen-branching enzyme gene was recorded through single-cell RNA sequencing in inguinal white adipocytes with active lipid synthesis [2].

In the experimental samples, the expression levels of both fatty acid synthesis enzymes and enzymes involved in lipolysis and beta-oxidation increased by approximately 1.5 to 2.5 times. This likely indicates that the oxidation of carbohydrates is no longer sufficient for meeting the energy needs of biosynthesis, forcing the adipocyte to utilize some of the energy stored in TAG by mobilizing lipolysis and actively oxidizing fatty acids. Consequently, intermittent short-term cold exposures without additional caloric compensation for their energy costs cause the adipocyte to enter a futile cycles mode—the simultaneous functioning of opposite processes: lipolysis and lipogenesis, beta-oxidation and fatty acid synthesis. These futile cycles reduce respiratory control, thus accelerating the energy metabolism of the adipocyte and increasing heat production. In cold-adapted mice with UCP1 gene knockout, the activity of the lipolysis–lipogenesis cycle in brown adipose tissue is higher compared to wild mice [49], meaning that the operation of this futile cycle is accompanied by energy dissipation which is equivalent or close in magnitude to UCP1-dependent thermogenesis.

Our data suggest the functioning of another futile cycle, in which the uncoupling protein UCP1 may theoretically participate. Among the mitochondrial proteins that increased their expression many times were acyl-CoA thioesterase2 (ACOT2) and acyl-CoA ligase of medium-chain fatty acids (ACSF2). ACOT2 hydrolyzes the CoA esters of long-chain fatty acids into free fatty acids and HSCoA. In our experiment, regular cold exposures resulted in an 11-fold increase in the expression levels of the enzyme ACOT2. The biological significance of the reaction catalyzed by ACOT2 is to prevent the depletion of mitochondrial pools of coenzyme A, NAD, and FAD in tissues with a high intensity of beta oxidation [50]. The anions of long-chain fatty acids released during the ACOT2 reaction can be utilized for lipogenesis, the synthesis of phospholipids, and steroids; can undergo elongation; and ultimately can re-enter the beta-oxidation pathway. In any of these scenarios, the fatty acid must first exit the mitochondria, as the acyl-CoA ligase of long-chain fatty acids is located in the cytoplasm. From a bioenergetics perspective, the synthesis of fatty acyl-CoA, its transport into the mitochondria, hydrolysis, and the transport of fatty acids out of the mitochondria can be regarded as a futile cycle. The reverse transport of fatty acid anions from the mitochondria can be conducted by UCP1 or the ADP/ATP translocase [51,52]. The mechanism of the uncoupling effect of UCP1 is not well understood. According to one of the two discussed models, UCP1 transports fatty acid anions into the intermembrane space, thereby accelerating the reverse carnitine-independent transport of protonated fatty acids into the mitochondria [51]. Medium-chain fatty acids form esters with coenzyme A directly in the mitochondrial matrix, penetrating through the inner membrane independently of carnitine in protonated form [53]. Therefore, they can potentially act as uncoupling agents [51]. A 9-fold increase in the expression of mitochondrial acyl-CoA ligase of medium-chain fatty acids (ACSF2) should facilitate their transport into the mitochondria. Thus, the mechanisms of mitochondrial uncoupling can participate in enhancing energy exchange and heat production, together with ATP-hydrolytic loads.

In the absence of an additional influx of nutrients, the structural reorganization of the mitochondrion and its high biological quality can be ensured by increasing the intensity of autophagy processes. This possibility is indicated by the increased expression of small GTPase Rab 1B and hexokinase 2 in our experimental samples. Rab 1B is a key protein for initiating the formation of autophagosome [54]. Hexokinase 2 binds and inhibits TORC1 to facilitate autophagy at low glucose-6-phosphate levels [23]. There was also a 6-fold increase in the expression of the COX 54 protein, which is supposed to inhibit proteasomal degradation of cellular proteins and prevent excessive destabilization of the proteome [55]. Mitochondriogenesis and autophagy can apparently be considered a futile cycle.

Particular attention should be paid to the cellular and systemic mechanisms that trigger and regulate the restructuring of metabolism in adipose tissue stimulated by cold. A nuclear thyroid hormone-responsive protein (THRSP) was identified among the up-regulated proteins in the experimental samples. THRSP is known to stimulate the transcription of lipogenic genes [56], fatty acid beta-oxidation, TCA cycle enzymes, and ETC respiratory complex proteins [57]. THRSP, like some other transcription factors involved in adipogenesis, is regulated by sirtuin 1 [58]. Although sirtuin 1 was not detected among the differentially expressed proteins, a significant five-fold increase in the expression of another participant in deacetylation processes, the enzyme acetyl-CoA synthetase (ACSA), in the experimental samples is noteworthy. Under the influence of stress factors, ACSA moves from the cytoplasm to the nucleus, participating in the recycling of the acetyl group in the processes of deacetylation and acetylation of proteins [59]. In addition, the second product of the ACSA reaction, AMP, activates AMP kinase, which is involved in signaling pathways that stimulate lipolysis and mitochondriogenesis [59].

## 4. Materials and Methods

### 4.1. Animals and Study Design of Intermittent Cold Exposures

Male Institute of Cancer Research (ICR) mice were purchased from the nursery of the State Research Center Vector (Koltsovo, Russia) at 6 weeks of age. The mice were kept at a temperature of 23 ± 2 °C and had ad libitum access to water and a balanced pelleted diet for laboratory rodents (10.5 MJ/kg metabolizable energy; 5% fat, 19% protein. BioPro, Novosibirsk, Russia). After a 1.5-week adaptation period, the mice were divided into two groups. The control group continued under the previously mentioned dietary and temperature conditions. The experimental group of mice was exposed to 6 °C for 6 h each day, 5 days a week, over a span of 16 weeks. Prior to the experiment, the duration of cold exposure was gradually increased, beginning with 3 h on the first day and adding an hour each day until reaching 6 h. The cold exposures started at 8.30–9.30 am. The choice of the morning time to start the cold exposures was based on similar studies, which indicated that transcriptional responses of adipose tissue to acute and chronic cold exposures are more pronounced during daylight hours when mice are inactive [60].

To eliminate the influence of cold-induced feed intake increase, the experimental mice were provided with a food amount equivalent to that consumed by the control animals. For this purpose, before daily feeding, the amount of food remaining from the previous feeding in the cages with control mice was weighed and its average consumption per animal was calculated. Based on this value, the required amount of food for the experimental animals was determined, which was equivalent to the amount of food consumed by the control mice. At the end of the experiment, animals that had been fasted for 4 h were sacrificed. Blood was collected using heparin, then centrifuged at 900× *g* at 4 °C for 15 min. The concentrations of glucose and triglycerides in blood plasma were determined using commercially available colorimetric assays, following the manufacturers’ protocols (Vital Development Corporation JSC, Saint Petersburg, Russia).

To evaluate the effect of the experimental regimen on the primary types of adipose tissue, in addition to the inguinal white adipose tissue (IWAT), the epididymal white adipose tissue (EWAT) and the interscapular brown adipose tissue (IBAT) were dissected, weighed, rinsed with cold saline, and used for measurements of total tissue protein. IWAT samples from 3 control and 4 experimental mice were selected for proteomic analysis.

### 4.2. Total Protein Assay in Adipose Tissues

The tissues were homogenized in a buffer containing 10 mM Tris-HCl and 1 mM ethylenediaminetetraacetic acid (EDTA, Sigma-Aldrich, Saint Louis, MO, USA), pH 7.2, supplemented with 1 mM phenylmethylsulfonyl fluoride (PMSF, Sigma-Aldrich, Saint Louis, MO, USA). The tissue/buffer ratio was 30–50 mg/0.5 mL for IBAT and 100–150 mg/0.5 mL for IWAT and EWAT. The homogenates were solubilized by adding 1% sodium dodecyl sulfate (SDS, Sigma-Aldrich, Saint Louis, MO, USA) and 0.44 M NaOH to a final concentration, and the protein content in them was determined by the Lowry method [61]. The relative content of total protein in tissue was calculated in µg/mg.

### 4.3. Proteomic Analysis of Inguinal Adipose Tissue

The samples were prepared for analysis in three chemical replicates, and each was analyzed twice. Tissues were cut into small pieces and lysed in a buffer solution containing 20 mM Tris pH 7.5, 2 mM EDTA, 150 mM NaCl, and 0.5% sodium deoxycholate. They were then homogenized on ice and centrifuged at 15,000× *g* for 10 min at 10 °C. The supernatants were stored at −80 °C. The concentration of proteins was determined using a NanoVue Plus spectrophotometer (GE Healthcare Life Sciences, Marlborough, MA, USA). For each analysis, an equivalent amount of a lysate sample with a protein content of 2 μg was taken. The samples were reduced with dithiothreitol, alkylated with iodoacetamide, and hydrolyzed with trypsin according to the protocols provided by the manufacturer of the reagents (Pierce Biotechnologies, Thermo Scientific, Waltham, MA, USA). Subsequently, the samples were cleaned using 10 μL pipette tips with a C18 phase from the same manufacturer, following its protocol.

The samples were dissolved in phase “A” (0.1% of formic acid), and 2 µg of each sample was injected into the Dionex UltiMate 3000 RSLC nano liquid chromatographer (Thermo Scientific, Waltham, MA, USA) using an Acclaim RSLC PepMap C18 separation column (15 cm length, 75 µm inner diameter, 2 µm particles). The solvent gradient increased from 0% to 40% of phase “B” (0.1% of formic acid in 80% acetonitrile) over 90 min while maintaining a constant flow rate of 200 nL/min. The Orbitrap Fusion mass spectrometer (Thermo Scientific, Waltham, MA, USA) operated in data-dependent mode, with scans of parent and fragment ions changing in a cycle of 4 s. Full scans were conducted at a resolution of 60,000 by the Orbitrap mass detector, and fragments generated by high-energy collision dissociation (HCD) were registered by the ion trap at a normal rate.

The raw data files were processed using MaxQuant version 1.6 software (Max Planck Institute for Biochemistry, Martinsried, Munich, Germany). The label-free quantification (LFQ) parameter was enabled. Unique and razor peptides were selected for protein quantification. Carbamidomethylation of cysteine was set as a fixed modification, while oxidation of methionine and N-terminal acetylation were designated as variable modifications. A protein search was performed against the SwissProt mouse database from the Uniprot website [62]. A protein false discovery rate (FDR) of 0.01 was set. Samples containing fewer than 200 proteins were excluded from further analysis. Proteins detected in less than 5 samples were not considered. The obtained LFQ intensities were regarded as relative indicators of protein expression across the sample groups.

Protein classification was conducted based on functional annotations using Gene Ontology (GO) for cellular components and biological processes in the PANTHER database (Protein Analysis through Evolutionary Relationships) [19]. For each classification category, the PANTHER overrepresentation test tool was employed to determine the actual number of genes in both the tested and reference lists, the expected number of genes in the tested lists, and the variance between the actual and expected numbers. The tested lists consisted of lists of up-regulated and down-regulated proteins in the experimental sample group, with the entire *Mus musculus* genome serving as the reference list.

### 4.4. Statistical Analysis

The results of measurements of body mass and tissue mass, as well as tissue protein, were presented as mean ± standard deviation. Statistical analyses were performed using Statistica 6 software with the non-parametric Mann–Whitney test. Differentially expressed proteins were determined by applying Welch’s t-test with a significance level of *p* < 0.05, corrected by Benjamini–Hochberg (FDR < 0.01). The statistical overrepresentation test was conducted using a Fisher exact test with the Benjamini–Hochberg FDR correction. Principal component analysis (PCA) was used to visualize the variance in protein expression between control and experimental samples.

## 5. Conclusions

Thus, the regime of regular cold exposures without compensating for their energy cost through eating, which begins during puberty, restricts the lipid expansion of adipocytes in adult animals. The results of the proteomic analysis indicate the activation of energy metabolism and the functioning of futile cycles, which are related to lipogenesis from carbohydrates. Regardless of their function, futile cycles decrease the need for thermoregulatory UCP1-dependent heat production. Further study on the role and mechanisms of mitochondrial uncoupling and UCP1-dependent thermogenesis in subcutaneous adipose tissue under intermittent short-term cold exposures requires analyzing cellular (basal, oligomycin-sensitive, norepinephrine-induced) respiration and utilizing sensitive immunochemical methods to identify the UCP1 protein in the dynamics of adaptation. The glucose homeostasis assessment using glucose tolerance and insulin tolerance tests is also needed for future studies.

## Figures and Tables

**Table 1:** Up-regulated proteins in the experimental (E) group compared with the control (C) group.

Protein ID	Gene Name	Protein Name	*p*-Value	Corrected *p*-Value *	LFQ^E^/LFQ^C^ Ratio
Q9Z2I0	LETM1	Mitochondrial proton/calcium exchanger protein	1.27 × 10^−3^	4.53 × 10^−3^	15.11
Q9Z0X1	AIFM1	Apoptosis-inducing factor 1, mitochondrial	9.68 × 10^−5^	6.53 × 10^−4^	14.5
P67778	PHB	Prohibitin	3.25 × 10^−8^	4.38 × 10^−6^	14.13
Q9D6Y9	GLGB	1,4-alpha-glucan-branching enzyme	1.60 × 10^−8^	3.23 × 10^−6^	14.09
O35129	PHB2	Prohibitin-2	4.18 × 10^−5^	4.03 × 10^−4^	12.63
Q9CR68	UCRI	Cytochrome b-c1 complex subunit Rieske, mitochondrial	3.62 × 10^−7^	2.01 × 10^−5^	12.19
Q9QZA0	CAH5B	Carbonic anhydrase 5B, mitochondrial	4.46 × 10^−5^	4.20 × 10^−4^	11.54
Q9QYR9	ACOT2	Acyl-coenzyme A thioesterase 2, mitochondrial	3.98 × 10^−7^	2.01 × 10^−5^	11.27
Q9DC69	NDUA9	NADH dehydrogenase [ubiquinone] 1 alpha subcomplex subunit 9, mitochondrial	1.73 × 10^−3^	5.90 × 10^−3^	11.18
P97450	ATP5J	ATP synthase-coupling factor 6, mitochondrial	2.51 × 10^−6^	5.65 × 10^−5^	9.74
Q8VCW8	ACSF2	Medium-chain acyl-CoA ligase ACSF2, mitochondrial	1.33 × 10^−4^	7.84 × 10^−4^	9.45
Q9D6J6	NDUV2	NADH dehydrogenase [ubiquinone] flavoprotein 2, mitochondrial	1.07 × 10^−4^	6.97 × 10^−4^	9.33
P06745	G6PI	Glucose-6-phosphate isomerase	9.27 × 10^−5^	6.36 × 10^−4^	8.59
Q91ZJ5	UGPA	UTP-glucose-1-phosphate uridylyl transferase	3.13 × 10^−7^	2.01 × 10^−5^	7.46
Q9DB20	ATPO	ATP synthase subunit O, mitochondrial	3.13 × 10^−3^	9.26 × 10^−3^	7.34
Q91V76	CK054	Ester hydrolase C11orf54 homolog	3.13 × 10^−3^	9.26 × 10^−3^	6.7
P12787	COX5A	Cytochrome c oxidase subunit 5A, mitochondrial	5.61 × 10^−6^	9.47 × 10^−5^	6.53
Q91YT0	NDUV1	NADH dehydrogenase [ubiquinone] flavoprotein 1, mitochondrial	2.36 × 10^−4^	1.23 × 10^−3^	6.24
Q9WUM5	SUCA	Succinate-CoA ligase [ADP/GDP-forming] subunit alpha, mitochondrial	1.55 × 10^−5^	1.96 × 10^−4^	6.1
O08528	HXK2	Hexokinase-2	2.26 × 10^−5^	2.52 × 10^−4^	5.92
Q9D1G1	RAB1B	Ras-related protein Rab-1B	3.62 × 10^−4^	1.73 × 10^−3^	5.65
P70404	IDHG1	Isocitrate dehydrogenase [NAD] subunit gamma 1, mitochondrial	4.13 × 10^−6^	7.40 × 10^−5^	5.46
Q62425	NDUA4	Cytochrome c oxidase subunit NDUFA4	3.42 × 10^−4^	1.67 × 10^−3^	5.46
Q9QXG4	ACSA	Acetyl-coenzyme A synthetase, cytoplasmic	8.35 × 10^−6^	1.30 × 10^−4^	5.32
Q64521	GPDM	Glycerol-3-phosphate dehydrogenase, mitochondrial	2.76 × 10^−6^	5.88 × 10^−5^	5.28
Q8BKZ9	ODPX	Pyruvate dehydrogenase protein X component, mitochondrial	5.20 × 10^−4^	2.29 × 10^−3^	5.01
P56391	CX6B1	Cytochrome c oxidase subunit 6B1	4.68 × 10^−4^	2.08 × 10^−3^	4.93
Q9DBB8	DHDH	Trans+G100dehydrogenase	5.71 × 10^−4^	2.46 × 10^−3^	4.9
Q91VD9	NDUS1	NADH-ubiquinone oxidoreductase 75 kDa subunit, mitochondrial	3.49 × 10^−6^	7.08 × 10^−5^	4.47
Q60597	ODO1	2-oxoglutarate dehydrogenase, mitochondrial	1.31 × 10^−5^	1.77 × 10^−4^	4.46
Q921G7	ETFD	Electron transfer flavoprotein-ubiquinone oxidoreductase, mitochondrial	4.99 × 10^−5^	4.39 × 10^−4^	4.38
Q8BMF4	ODP2	Dihydrolipoyl lysine-residue acetyltransferase component of pyruvate dehydrogenase complex, mitochondrial	1.44 × 10^−6^	4.17 × 10^−5^	4.34
P47934	CACP	Carnitine O-acetyltransferase	8.30 × 10^−5^	6.01 × 10^−4^	4.2
P12382	PFKAL	ATP-dependent 6-phosphofructokinase, liver type	7.79 × 10^−5^	5.84 × 10^−4^	4.07
Q9DCT2	NDUS3	NADH dehydrogenase [ubiquinone] iron-sulfur protein 3, mitochondrial	5.76 × 10^−5^	4.58 × 10^−4^	3.81
P35486	ODPA	Pyruvate dehydrogenase E1 component subunit alpha, somatic form, mitochondrial	2.47 × 10^−5^	2.63 × 10^−4^	3.78
Q60932	VDAC1	Voltage-dependent anion-selective channel protein 1	2.46 × 10^−7^	2.01 × 10^−5^	3.71
Q9D2G2	ODO2	Dihydrolipoyllysine-residue succinyltransferase component of 2-oxoglutarate dehydrogenase complex, mitochondrial	6.63 × 10^−4^	2.71 × 10^−3^	3.6
Q91VR2	ATPG	ATP synthase subunit gamma, mitochondrial	6.27 × 10^−4^	2.62 × 10^−3^	3.55
Q8JZU2	TXTP	Tricarboxylate transport protein, mitochondrial	1.43 × 10^−6^	4.17 × 10^−5^	3.38
Q91ZA3	PCCA	Propionyl-CoA carboxylase alpha chain, mitochondrial	3.32 × 10^−3^	9.67 × 10^−3^	3.29
Q00612	G6PD1	Glucose-6-phosphate 1-dehydrogenase X	4.99 × 10^−5^	4.39 × 10^−4^	3.09
Q8CGN5	PLIN1	Perilipin-1	1.23 × 10^−4^	7.80 × 10^−4^	2.99
P50544	ACADV	Very long-chain specific acyl-CoA dehydrogenase, mitochondrial	8.94 × 10^−7^	3.62 × 10^−5^	2.97
Q9ET01	PYGL	Glycogen phosphorylase, liver form	7.49 × 10^−4^	2.97 × 10^−3^	2.97
P52825	CPT2	Carnitine O-palmitoyl transferase 2, mitochondrial	2.16 × 10^−3^	6.99 × 10^−3^	2.76
P06801	MAOX	NADP-dependent malic enzyme	2.30 × 10^−5^	2.52 × 10^−4^	2.74
Q8BMS1	ECHA	Trifunctional enzyme subunit alpha, mitochondrial	1.07 × 10^−5^	1.58 × 10^−4^	2.68
P97807	FUMH	Fumarate hydratase, mitochondrial	3.98 × 10^−5^	3.94 × 10^−4^	2.59
Q9D051	ODPB	Pyruvate dehydrogenase E1 component subunit beta, mitochondrial	8.30 × 10^−6^	1.30 × 10^−4^	2.51
P51881	ADT2	ADP/ATP translocase 2	1.10 × 10^−6^	4.05 × 10^−5^	2.5
Q99MN9	PCCB	Propionyl-CoA carboxylase beta chain, mitochondrial	2.33 × 10^−3^	7.42 × 10^−3^	2.49
Q91V92	ACLY	ATP-citrate synthase	4.67 × 10^−10^	1.90 × 10^−7^	2.46
Q99JY0	ECHB	Trifunctional enzyme subunit beta, mitochondrial	3.48 × 10^−5^	3.59 × 10^−4^	2.44
P00405	COX2	Cytochrome c oxidase subunit 2	5.85 × 10^−5^	4.58 × 10^−4^	2.38
P51174	ACADL	Long-chain specific acyl-CoA dehydrogenase, mitochondrial	9.29 × 10^−4^	3.58 × 10^−3^	2.38
Q07417	ACADS	Short-chain specific acyl-CoA dehydrogenase, mitochondrial	5.88 × 10^−5^	4.58 × 10^−4^	2.37
Q9D9V3	ECHD1	Ethylmalonyl-CoA decarboxylase	8.08 × 10^−5^	5.95 × 10^−4^	2.35
P54310	LIPS	Hormone-sensitive lipase	1.09 × 10^−3^	4.07 × 10^−3^	2.35
Q9D6R2	IDH3A	Isocitrate dehydrogenase [NAD] subunit alpha, mitochondrial	1.20 × 10^−5^	1.68 × 10^−4^	2.28
Q61425	HCDH	Hydroxyacyl-coenzyme A dehydrogenase, mitochondrial	1.04 × 10^−3^	3.94 × 10^−3^	2.17
Q9CZU6	CISY	Citrate synthase, mitochondrial	1.36 × 10^−5^	1.78 × 10^−4^	2.12
Q9D0M3	CY1	Cytochrome c1, heme protein, mitochondrial	1.27 × 10^−4^	7.84 × 10^−4^	2.11
Q9CZ13	QCR1	Cytochrome b-c1 complex subunit 1, mitochondrial	9.15 × 10^−5^	6.36 × 10^−4^	2.1
Q5SWU9	ACACA	Acetyl-CoA carboxylase 1	4.20 × 10^−6^	7.40 × 10^−5^	2.09
Q8K2B3	SDHA	Succinate dehydrogenase [ubiquinone] flavoprotein subunit, mitochondrial	6.89 × 10^−4^	2.77 × 10^−3^	2.04
P14152	MDHC	Malate dehydrogenase, cytoplasmic	1.32 × 10^−3^	4.65 × 10^−3^	2.03
Q99KI0	ACON	Aconitate hydratase, mitochondrial	1.65 × 10^−5^	2.02 × 10^−4^	2
Q62264	THRSP	Thyroid hormone-inducible hepatic protein	3.68 × 10^−4^	1.73 × 10^−3^	1.99
Q9DCD0	6PGD	6-phosphogluconate dehydrogenase, decarboxylating	1.28 × 10^−6^	4.17 × 10^−5^	1.98
Q9CQ62	DECR	2,4-dienoyl-CoA reductase, mitochondrial	3.18 × 10^−4^	1.57 × 10^−3^	1.94
P05064	ALDOA	Fructose-bisphosphate aldolase A	4.84 × 10^−7^	2.18 × 10^−5^	1.92
P40142	TKT	Transketolase	3.89 × 10^−7^	2.01 × 10^−5^	1.89
P62897	CYC	Cytochrome c, somatic	1.34 × 10^−3^	4.68 × 10^−3^	1.89
O08749	DLDH	Dihydrolipoyl dehydrogenase, mitochondrial	2.10 × 10^−6^	5.02 × 10^−5^	1.88
P52480	KPYM	Pyruvate kinase PKM	2.11 × 10^−6^	5.02 × 10^−5^	1.84
Q8BWT1	THIM	3-ketoacyl-CoA thiolase, mitochondrial	2.09 × 10^−6^	5.02 × 10^−5^	1.83
P38647	GRP75	Stress-70 protein, mitochondrial	1.57 × 10^−4^	8.59 × 10^−4^	1.81
Q05920	PYC	Pyruvate carboxylase, mitochondrial	2.77 × 10^−4^	1.42 × 10^−3^	1.78
P09411	PGK1	Phosphoglycerate kinase 1	5.91 × 10^−5^	4.58 × 10^−4^	1.77
P45952	ACADM	Medium-chain specific acyl-CoA dehydrogenase, mitochondrial	3.64 × 10^−4^	1.73 × 10^−3^	1.75
P42125	ECI1	Enoyl-CoA delta isomerase 1, mitochondrial	1.67 × 10^−3^	5.72 × 10^−3^	1.7
Q9DB77	QCR2	Cytochrome b-c1 complex subunit 2, mitochondrial	2.69 × 10^−3^	8.25 × 10^−3^	1.69
Q9DBJ1	PGAM1	Phosphoglycerate mutase 1	4.03 × 10^−4^	1.83 × 10^−3^	1.61
P08249	MDHM	Malate dehydrogenase, mitochondrial	3.97 × 10^−6^	7.40 × 10^−5^	1.52
P56480	ATPB	ATP synthase subunit beta, mitochondrial	5.85 × 10^−5^	4.58 × 10^−4^	1.46
P13707	GPDA	Glycerol-3-phosphate dehydrogenase [NAD^(+)^], cytoplasmic	1.81 × 10^−3^	6.05 × 10^−3^	1.46
P41216	ACSL1	Long-chain-fatty-acid-CoA ligase 1	1.10 × 10^−3^	4.07 × 10^−3^	1.45
P19096	FAS	Fatty acid synthase	1.75 × 10^−3^	5.91 × 10^−3^	1.44
P20108	PRDX3	Thioredoxin-dependent peroxide reductase, mitochondrial	5.60 × 10^−4^	2.44 × 10^−3^	1.43
P16858	G3P	Glyceraldehyde-3-phosphate dehydrogenase	3.05 × 10^−3^	9.16 × 10^−3^	1.43
Q03265	ATPA	ATP synthase subunit alpha, mitochondrial	1.34 × 10^−4^	7.84 × 10^−4^	1.42
Q9DCW4	ETFB	Electron transfer flavoprotein subunit beta	3.29 × 10^−3^	9.66 × 10^−3^	1.4
Q05816	FABP5	Fatty acid-binding protein 5	7.89 × 10^−4^	3.10 × 10^−3^	1.36
P63038	CH60	60 kDa heat shock protein, mitochondrial	2.67 × 10^−3^	8.25 × 10^−3^	1.3
P48962	ADT1	ADP/ATP translocase 1	1.30 × 10^−4^	7.84 × 10^−4^	Presented in experimental group only
Q9Z2Z6	MCAT	Mitochondrial carnitine/acylcarnitine carrier protein	1.46 × 10^−4^	8.20 × 10^−4^	
Q791V5	MTCH2	Mitochondrial carrier homolog 2	1.57 × 10^−4^	8.59 × 10^−4^	
Q6P3A8	ODBB	2-oxoisovalerate dehydrogenase subunit beta, mitochondrial	8.57 × 10^−4^	3.34 × 10^−3^	
Q8BJ56	PLPL2	Patatin-like phospholipase domain-containing protein 2	9.55 × 10^−4^	3.65 × 10^−3^	
Q99LC3	NDUAA	NADH dehydrogenase [ubiquinone] 1 alpha subcomplex subunit 10, mitochondrial	1.23 × 10^−3^	4.47 × 10^−3^	

* Benjamini–Hochberg corrected *p*-value.

**Table 2:** Down-regulated proteins in the experimental (E) group compared with the control (C) group.

Protein ID	Gene Name	Protein Name	*p*-Value	Corrected *p*-Value *	LFQ^C^/LFQ^E^ Ratio
Q91X72	HEMO	Hemopexin	1.09 × 10^−5^	1.58 × 10^−4^	1.911
P19324	SERPH	Serpin H1	1.79 × 10^−5^	2.13 × 10^−4^	3.432
Q00896	A1AT3	Alpha-1-antitrypsin 1-3	1.90 × 10^−5^	2.20 × 10^−4^	2.960
P51885	LUM	Lumican	3.55 × 10^−5^	3.59 × 10^−4^	1.360
P20029	BIP	Endoplasmic reticulum chaperone BiP	4.74 × 10^−5^	3.90 × 10^−4^	1.567
P21614	VTDB	Vitamin D-binding protein	5.21 × 10^−5^	4.51 × 10^−4^	2.086
P22599	A1AT2	Alpha-1-antitrypsin 1-2	5.99 × 10^−5^	5.13 × 10^−4^	2.663
P08113	ENPL	Endoplasmin	9.06 × 10^−5^	5.74 × 10^−4^	1.860
P11499	HS90B	Heat shock protein HSP 90-beta	8.88 × 10^−5^	6.35 × 10^−4^	1.447
P27773	PDIA3	Protein disulfide-isomerase A3	9.92 × 10^−5^	6.96 × 10^−4^	1.744
P40124	CAP1	Adenylyl cyclase-associated protein 1	1.10 × 10^−4^	7.57 × 10^−4^	1.560
O89053	COR1A	Coronin-1A	1.20 × 10^−4^	8.18 × 10^−4^	1.495
P13020	GELS	Gelsolin	1.31 × 10^−4^	8.79 × 10^−4^	2.033
Q60605	MYL6	Myosin light polypeptide 6	1.41 × 10^−4^	9.40 × 10^−4^	1.557
P06728	APOA4	Apolipoprotein A-IV	1.52 × 10^−4^	1.00 × 10^−3^	3.061
P09405	NUCL	Nucleolin	1.62 × 10^−4^	1.06 × 10^−3^	1.345
Q921I1	TRFE	Serotransferrin	1.73 × 10^−4^	1.12 × 10^−3^	1.754
P07724	ALBU	Albumin	1.83 × 10^−4^	1.18 × 10^−3^	1.708
P18760	COF1	Cofilin-1	1.93 × 10^−4^	1.25 × 10^−3^	1.370
O08677	KNG1	Kininogen-1	2.04 × 10^−4^	1.31 × 10^−3^	1.526
O88569	ROA2	Heterogeneous nuclear ribonucleoproteins A2/B1	2.14 × 10^−4^	1.37 × 10^−3^	1.478
P09103	PDIA1	Protein disulfide-isomerase	2.25 × 10^−4^	1.43 × 10^−3^	1.542
P63158	HMGB1	High mobility group protein B1	2.35 × 10^−4^	1.49 × 10^−3^	3.579
P63101	1433Z	14-3-3 protein zeta/delta	2.46 × 10^−4^	1.55 × 10^−3^	1.413
P07901	HS90A	Heat shock protein HSP 90-alpha	2.56 × 10^−4^	1.61 × 10^−3^	1.343
P63017	HSP7C	Heat shock cognate 71 kDa protein	2.67 × 10^−4^	1.67 × 10^−3^	1.235
P60710	ACTB	Actin, cytoplasmic 1	2.77 × 10^−4^	1.73 × 10^−3^	1.407
Q8BH61	F13A	Coagulation factor XIII A chain	2.88 × 10^−4^	1.80 × 10^−3^	2.652
P29699	FETUA	Alpha-2-HS-glycoprotein	2.98 × 10^−4^	1.86 × 10^−3^	2.498
O70423	AOC3	Membrane primary amine oxidase	3.09 × 10^−4^	1.92 × 10^−3^	1.375
P08752	GNAI2	Guanine nucleotide-binding protein G(i) subunit alpha-2	3.19 × 10^−4^	1.98 × 10^−3^	1.309
P16015	CAH3	Carbonic anhydrase 3	3.30 × 10^−4^	2.04 × 10^−3^	1.424
P17742	PPIA	Peptidyl-prolyl cis-trans isomerase A	3.40 × 10^−4^	2.10 × 10^−3^	1.374
Q00623	APOA1	Apolipoprotein A-I	2.13 × 10^−3^	6.96 × 10^−3^	0.447
Q9Z0F7	SYUG	Gamma-synuclein	3.50 × 10^−4^	2.16 × 10^−3^	Presented in control group only
P01872	IGHM	Immunoglobulin heavy constant mu	3.61 × 10^−4^	2.22 × 10^−3^	16.667
Q91YQ5	RPN1	Dolichyl-diphosphooligosaccharide-protein glycosyltransferase subunit 1	3.71 × 10^−4^	2.29 × 10^−3^	10.145
Q61147	CERU	Ceruloplasmin	3.82 × 10^−4^	2.35 × 10^−3^	10.653

* Benjamini–Hochberg corrected *p*-value.

## Data Availability

The mass spectrometry results dataset is available on request from the corresponding author (Y.G.).

## Abbreviations

The following abbreviations are used in this manuscript:

ICE	Intermittent cold exposure
IWAT	Inguinal white adipose tissue
EWAT	Epididymal white adipose tissue
IBAT	Interscapular brown adipose tissue
TCA	Tricarboxylic acid
ICR	Institute of Cancer Research
EDTA	Ethylenediaminetetraacetic acid
PMSF	Phenylmethylsulfonyl fluoride
SDS	Sodium dodecyl sulfate
TBST	Tris-buffered saline solution containing Tween 20
BSA	Bovine serum albumin
LFQ	Label-free quantification
FDR	False discovery rate
PCA	Principal component analysis
GO	Gene Ontology
PANTHER	Protein Analysis through Evolutionary Relationships
TAG	Triacylglycerol
ETC	Electron transport chain
EPR	Endoplasmic reticulum
ECM	Extracellular matrix
